# Fitness of *Mythimna separata* (Lepidoptera: Noctuidae) on Cultivated Wheat and a Weed, Wild Oat (*Avena fatua*), and Its Implications for Pest Management

**DOI:** 10.3390/biology13121037

**Published:** 2024-12-11

**Authors:** Qinjian Pan, Junyu Shen, Lvming Su, Zexiang Nie, Ikkei Shikano, Tong-Xian Liu, Lin Chen

**Affiliations:** 1 Joint International Research Laboratory of Agriculture and Agri-Product Safety, The Ministry of Education of China, Institutes of Agricultural Science and Technology Development, Yangzhou University, Yangzhou 225091, China; 2 College of Agriculture, Yangzhou University, Yangzhou 225091, China; 3 College of Plant Protection, Yangzhou University, Yangzhou 225091, China; 4 Department of Plant and Environmental Protection Sciences, University of Hawai’i at Mānoa, Honolulu, HI 96822, USA; 5 Institute of Entomology, Guizhou University, Guiyang 550025, China; 6 Institute of Plant Health and Medicine, Guizhou University, Guiyang 550025, China

**Keywords:** oriental armyworm, plant preference, development, oviposition

## Abstract

*Mythimna separata* (Walker), a significant migratory pest in many Asian countries, can cause severe damage to wheat crops. Understanding whether wild oat can serve as an alternate host is important for informing predictive models of *M. separata* infestation levels in wheat fields and can improve pest and weed management strategies. We first conducted both choice and no-choice experiments and found that *M. separata* readily laid eggs on both wheat and wild oat, with no significant oviposition preference. However, newly hatched larvae exhibited a strong preference for wheat, with 53.34% more larvae feeding on wheat compared to wild oat. Development time from egg to adult emergence was significantly shorter in *M. separata* fed on wheat than on wild oat, although the durations of the larval and pupal stages varied depending on the host plant. Despite these differences in development time, host plants did not significantly affect larval survival, pupal weight, fecundity, and adult longevity. The mean number of eggs laid by females that fed on wheat and wild oat were 1202.12 and 1270.54 eggs/female, and larval survival rates were 81% and 84%, respectively. Our findings suggest that wild oat can serve as a suitable host for *M. separata* and may act as a refuge when wheat fields are treated with insecticides. The high larval preference for wheat over wild oat indicates that *M. separata* on wild oat may easily disperse to wheat, complicating pest control and highlighting the need to include wild oat in pest management strategies.

## Simple Summary

*Mythimna separata* (Walker) is a migratory pest that is widely distributed in many Asian countries, including China. Though it is known to have a wide range of hosts, few studies have assessed the insect’s fitness on various host plants, especially weeds. Understanding the fundamental biology and ecology of this pest is crucial for developing effective control methods. The present study examined the oviposition preference of female adults, larval preference, development, reproduction, and survival of *M. separata* on cultivated wheat and a weed, wild oat (*Avena fatua*). We found that although *M. separata* females showed no oviposition preference on both plants, the larvae exhibited a strong preference for wheat compared to wild oat. *M. separata* reared on wheat had a shorter development time from egg to adult emergence compared to wild oat. *M. separata* reared on wild oat achieved high larval survival, adult longevity, and fecundity, which were not significantly different from those reared on wheat. Our results highlight that wild oat could serve as a bridge host for *M. separata* to complete its life cycle and sustain populations when cultivated hosts such as wheat are absent.

## 1. Introduction

The oriental armyworm, *Mythimna separata* (Walker) (Lepidoptera: Noctuidae), is a notorious agriculture pest that is widely distributed in China as well as other Asian countries [1,2]. There are numerous published reports implicating *M. separata* in causing severe damage in fields of wheat, sorghum, and millet in different regions [2,3]. In China, *M. separata* has been found in all regions except Tibet and Xinjiang. Due to its short life cycle and high adaptability, they cause severe regional infestation and present a constant threat to farmers [4]. Importantly, this pest possesses strong dispersal and migration ability, leading to continuous infestations from 2017 to 2020 in northeast China [2,5]. Another factor that makes *M. separata* a serious pest is its capacity to evolve insecticide resistance [6,7]. In 2020, *M. separata* was classified as a first-class crop disease and insect pest by the Chinese Ministry of Agriculture and Rural Affairs [8]. Therefore, it is crucial to develop integrated pest management strategies to mitigate crop yield losses caused by *M. separata*.

*Mythimna separata* larvae are polyphagous, capable of feeding on more than 95 plant species across 18 plant families, the majority of which belong to Gramineae, Brassicaceae, Cucurbitaceae, and Leguminosae [9]. While their preferred host plants include numerous cultivated crops, including wheat, many weed species in these families could serve as alternative hosts that allow *M. separata* to evade pesticide applications on the crops and/or to bridge the pest’s life cycle in between crop cycles. The quality of host plants influences a wide range of insect fitness parameters, including survival, development rate, adult longevity, and fecundity [10,11]. Studies of other major lepidopteran pests of agricultural crops, such as the diamondback moth (*Plutella xylostella*) and the fall armyworm (*Sprodoptera frugiperda*), have demonstrated that these pests will readily use less suitable hosts that are available when their preferred hosts are scarce [12,13,14,15].

Wheat is the most widely cultivated crop, with an annual growing area spanning 217 million hectares [16]. It is crucial for feeding the ever-increasing human population and enhancing food security by providing 20% of global food calories and proteins [17]. Weeds can cause complete failure of wheat production [18]. Wild oat, *Avena fatua*, is considered one of the world’s 10 worst weeds in diverse agroecosystems. Its infestation directly or indirectly contributes to yield losses of up to 70% in cereal crops [19,20]. In wheat fields, wild oats can cause yield losses of up to 40% at densities of around 250 plants per square meter [21]. Since wild oat is a gramineous plant, it could be a suitable host plant for *M. separata*. *Mythimna separata* development is known to be significantly influenced by host plant species as well as environmental conditions [4,22,23]. Though wild oats are a common weed in wheat fields, their capacity to harbor *M. separata* and supplement their development is unknown. Moreover, *M. separata* is frequently reported to be a voracious pest of wheat [24,25], but *M. separata* development and reproduction on wheat has not been empirically measured. Here, we first assessed *M. separata* oviposition preference and larval feeding preference for wheat and wild oat. Then, we examined the impact of the two host plants on larval survival, development, and the fecundity of the resulting adults. Our findings reveal the extent to which a common weed could be harboring *M. separata* populations in wheat agroecosystems, which has important implications for the management of this key pest.

## 2. Materials and Methods

### 2.1. Host Plants

Wheat (*Triticum aestivum* var. Xinong 979) seeds were purchased from Jindao Seeds (Yangling, Shaanxi, China) and were not treated with any fungicides or insecticides. The seeds of wild oat *Avena fatua* were collected from unmanaged areas surrounding wheat fields (Yangling, Shaanxi, China). All seeds were planted in 180 mL plastic pots containing potting medium and kept in a climate-controlled room at 25 ± 1 °C (day), 22 ± 1 °C (night), and 65% RH, with a photoperiod of 14:10 (L:D) h. The seedlings were maintained without insecticide applications and used in all experiments when they reached 10–12 cm in height.

### 2.2. Insects

Pupae of *M. separata* were collected from a wheat field in Yangling, Shannxi, China (108.07° E, 34.28° N). Pupae were maintained in a climate-controlled insect rearing room until adult emergence. Newly emerged adults were provided with a 10% honey solution as an adult diet to supplement nutrition for adults. Individual mating pairs were placed in plastic cups (30 cm in diameter and 30 cm in height) covered with plastic film. Egg masses were collected, and the resulting newly hatched larvae were transferred to wheat or wild oat seedlings to produce lineages reared on each of the host plants. *M. separata* were maintained on wheat and wild oat in their respective nylon gauze cages (200 mesh size, 60 cm × 60 cm × 60 cm) for three generations before being used in the development and reproduction experiments. *M. separata* larvae reared on a wheat germ and casein-based artificial diet, as described by Jia et al. [26], were also maintained for three generations to produce an artificial diet population. Newly hatched larvae from this artificial diet population were used to test their feeding preference.

### 2.3. Oviposition Preference

**Figure 1:**
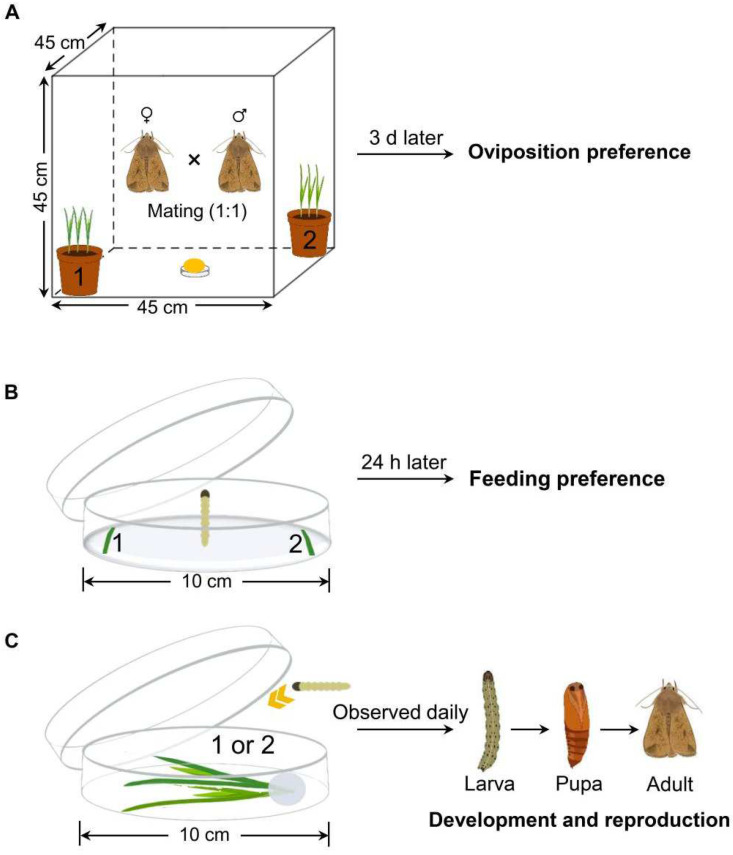
Schematic of the experimental method. (**A**) Oviposition preference, (**B**) feeding preference, and (**C**) development and reproduction of *Mythimna separata*.

To eliminate the influence of prior larval diet on host plant choice, *M. separata* pupae were collected from the artificial diet population to evaluate female oviposition preference. A pair of virgin adults (♀:♂ = 1:1) emerging within 24 h was released and allowed to mate in a mesh-covered cage (45 × 45 × 45 cm). A 3 cm diameter Petri dish containing a cotton ball soaked in adult diet was placed in the center of the cage. Three combinations of two-choice tests (wheat vs. wheat, wild oat vs. wild oat, wheat vs. wild oat) were set up by placing two 180 mL plastic pots containing the plants on opposite sides of the cage as oviposition substrates. A total of 30 mating pairs of virgin adults were prepared for the oviposition test for each plant combination. The distance between the two plants (plant 1 and plant 2) was always from one corner of the cage bottom to the diagonally opposite corner (Figure 1A). The time of the first egg being laid in each cage was checked daily, and from that day on, the number of eggs laid during three days of egg laying was counted on both plants.

### 2.4. Feeding Preference

*Mythimna separata* larvae from the artificial diet population were given a choice between wheat and wild oat leaves. The artificial diet population was used to limit the influence of parental diet on host plant choice. A piece of leaf (2 cm in length) from each plant was placed in a 10 cm diameter Petri dish, which was lined with 1% agar. The piece of wheat leaf was placed 8 cm apart from the piece of wild oat leaf (Figure 1B). A single newly hatched *M. separata* larva was released in the midpoint between the two leaves and kept at 25 ± 1 °C (day), 22 ± 1 °C (night), 65% RH, and 14:10 (L:D) for 24 h to allow the larva to choose its preferred food. A total of 20 replicate dishes were produced, and the number of larvae found on wheat or wild oat was recorded. The percentage of larvae that chose each host was calculated (i.e., the number of larvae that chose wheat or wild oat divided by 20 larvae). The experiment was performed three times to perform a statistical analysis of larval host preference.

### 2.5. Development of Immature Stages

Eggs laid by the wheat and wild oat populations, within 24 h of laying, were individually transferred to separate Petri dishes (10 cm diameter) containing fresh leaves of their respective host plants (Figure 1C). The petioles of the detached leaves were inserted into a water-soaked cotton ball to keep them fresh. A total of 120 Petri dishes, each containing one egg, were prepared: 100 for assessing fitness parameters on each host plant and 20 as spare samples for pairing adults for mating. Leaves were replaced daily until the larvae died or pupated. The durations of the egg, larval, and pupal stages were recorded. Thirty pupae from each plant treatment were randomly selected to be weighed on a microbalance. *M. separata* sex was identified at the pupal stage. Data on developmental measures from individuals that died were excluded from statistical analyses.

### 2.6. Adult Longevity and Female Fecundity

A mating pair of *M. separata* moths (<24 h since emergence), which were reared on wheat or wild oat as larvae, was placed in a plastic container (15 cm in diameter and 30 cm in depth) covered with plastic film. A total of 41 female and 40 male adults emerged from larvae fed on wheat, while 42 female and 42 male adults emerged from larvae fed on wild oat. One male adult that emerged from the spare larvae fed on wheat was used to mate with a female that was fed wheat during the experiment. A piece of paper towel sprayed with 10% honey solution was hung in each container to supply adults with nutrition and oviposition substrate. The containers were monitored daily to assess adult longevity, pre-oviposition period (i.e., from adult emergence to the date of the first egg being laid), and the number of eggs laid until the death of both moths. Females that did not lay any eggs were excluded from statistical analyses.

### 2.7. Statistical Analysis

The results of oviposition preference, feeding preference, durations of development (i.e., egg, larval, and pupal) stages, pupal weight, pre-oviposition period, and female fecundity were analyzed with a Student’s *t*-test at *α* = 0.05 to determine significant differences between the two populations. Differences in adult longevity were analyzed by two-way analysis of variance (ANOVA), and Duncan’s multiple range test was used to determine significant differences between treatments (*p* < 0.05). Larval mortality was assessed by Kaplan–Meier survival analysis, followed by pairwise comparisons performed with log-rank tests.

## 3. Results

### 3.1. Oviposition Preference

**Figure 2:**
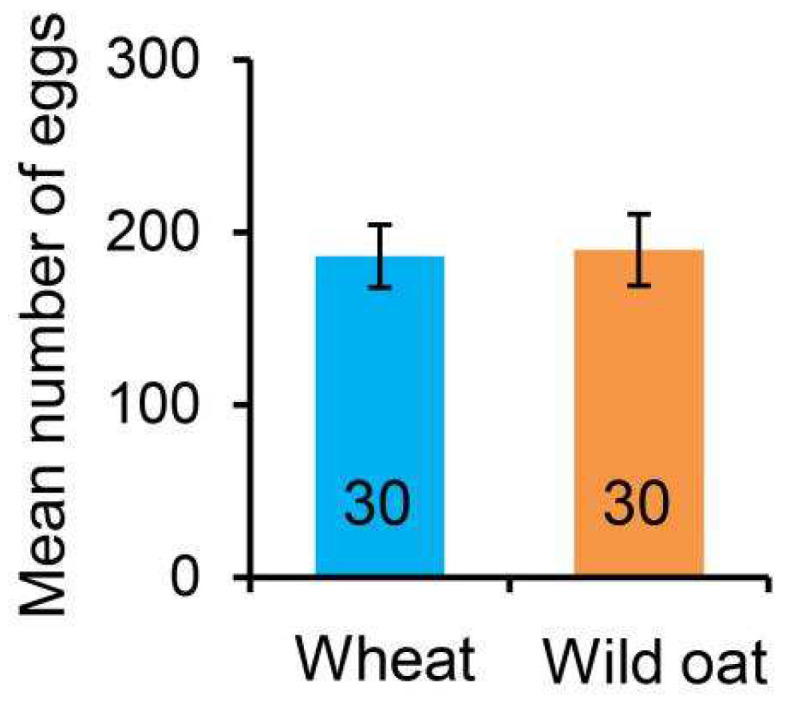
The mean number of eggs (±SE) laid by individual *Mythimna separata* females during a 3-day oviposition period when given a choice between wheat and wild oat. Numbers in the bars indicate sample sizes.

To evaluate the oviposition preference of *M. separata* females on wheat and wild oat, two-choice plant combinations of wheat/wheat, wild oat/wild oat, and wheat/wild oat were used to test the total number of eggs laid by individual females in three days. The plant combinations of wheat/wheat and wild oat/wild oat were employed as controls to evaluate directional bias in oviposition choice. The average number of eggs laid on the wheat/wheat combination was 255.17 and 238.50 (*t* = −0.468, *p* = 0.641), while the counts for the wild oat/wild oat choice were 158.07 and 187.93 (*t* = −1.843, *p* = 0.070). When the *M. separata* had a choice between wheat and wild oat, they exhibited no significant preference, with an average of 186.20 eggs on wheat and 189.60 eggs on wild oat (Figure 2: *t* = −0.124, *p* = 0.902).

### 3.2. Feeding Preference

**Figure 3:**
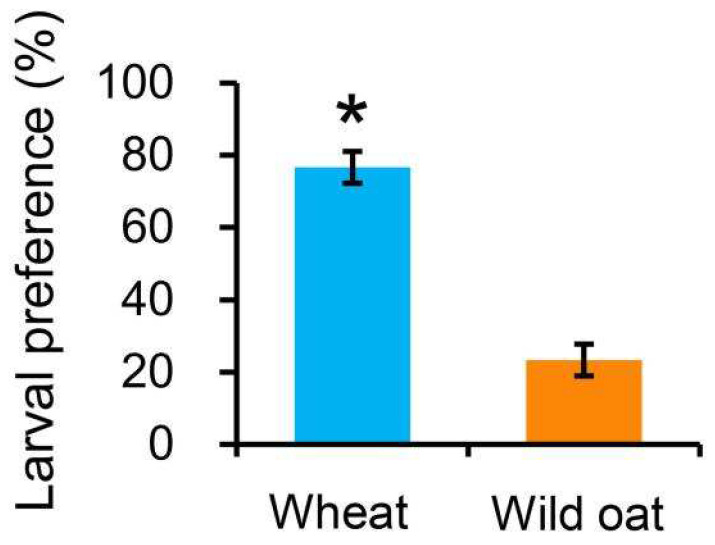
The percentage of newly hatched *Mythimna separata* larvae found on wheat or wild oat leaves after 24 h in a two-choice experiment. Values are means ± SE. Asterisk represents statistically significant difference between wheat and wild oat (*p* < 0.05).

Significantly, more *M. separata* larvae chose to feed on wheat leaves compared to wild oat leaves (Figure 3; *t* = 8.552, *p* = 0.001), with 76.67% of larvae choosing wheat leaves and 23.33% of larvae choosing wild oat leaves.

### 3.3. Development and Survival of Immature Stages

Eggs laid by the *M. separata* maintained for three generations on wheat took significantly longer to hatch than eggs laid by the wild oat-reared *M. separata* (eggs laid on their respective host plants) (Table 1; *t* = 4.069, *p* = 0.0001). The duration of larval development on wheat (15.53 d) was significantly shorter than development on wild oat (16.13 d) (*t* = −2.516, *p* = 0.013). This prolonged development on wild oat was mainly attributable to a significantly longer final (sixth) instar on wild oat than wheat (*t* = −5.993, *p* = 0.0001). The durations of first (*t* = −1.746, *p* = 0.0830), second (*t* = 0.662, *p* = 0.509), and fourth (*t* = −0.810, *p* = 0.4190) instar were not significantly different on the two host plants, and the fifth instar was longer on wheat than wild oat (*t* = 5.145, *p* = 0.0001. The pre-pupal period was shorter in larvae that developed on wheat than wild oat, though not significant (*t* = −1.941, *p* = 0.054). *M. separata* that developed on wheat emerged from pupation faster than those that developed on wild oat (*t* = −13.687, *p* = 0.0001). The sex ratio was approximately 1:1 for both treatments. Altogether, *M. separata* development from egg to adult emergence was significantly faster on wheat (29.20 d) than wild oat (32.65 d) (*t* = −11.979, *p* = 0.0001).

**Figure 4:**
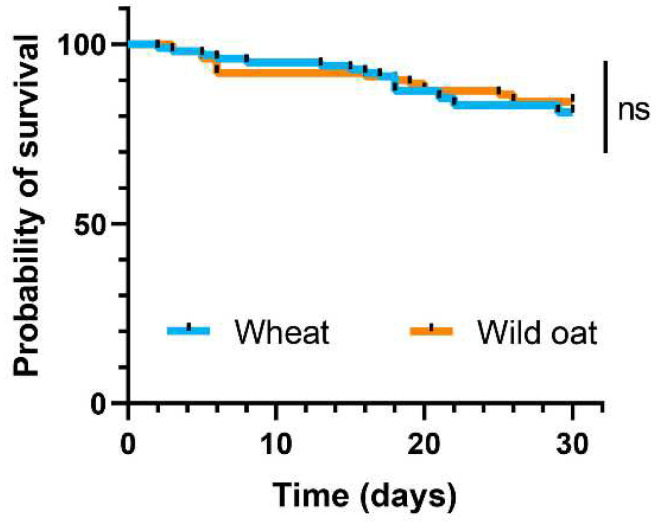
Survivorship of *Mythimna separata* larvae, shown as Kaplan–Meier estimates over 30 days, was compared using log-rank tests to assess the survival differences between larvae fed on leaves of wheat and wild oat. In the figure, ‘ns’ indicates a non-significant difference.

Survival from egg to adult emergence was high on both wheat (81%) and wild oat (84%) and not significantly different between the two plants (Figure 4; *χ*^2^ = 0.270, *p* = 0.603).

### 3.4. Adult Parameters

**Figure 5:**
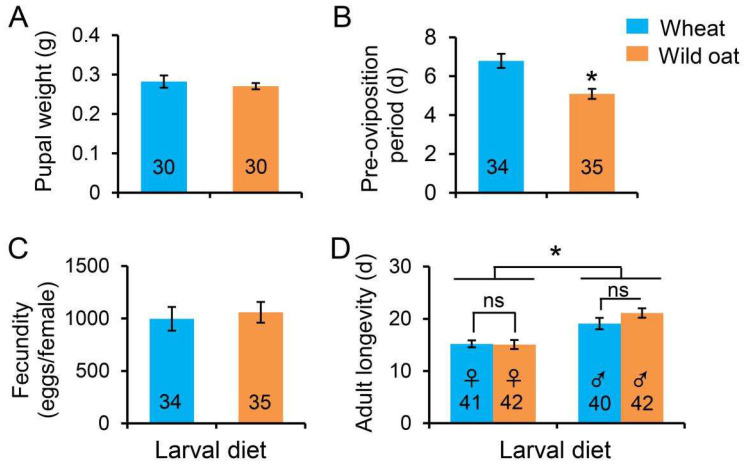
(**A**) Pupal weight, (**B**) pre-oviposition period, (**C**) fecundity, and (**D**) adult longevity of *Mythimna separata* fed leaves of either wheat or wild oat. Values are means ± SE. Asterisks represent statistically significant differences between treatments (*p* < 0.05), ‘ns’ indicates a non-significant difference. Numbers in the bars indicate sample sizes.

There was no significant difference in pupal weight (i.e., proxy for adult size) between larvae fed wheat and wild oat (Figure 5A; *t* = 0.670, *p* = 0.506). The pre-oviposition period was significantly longer when larvae developed on wheat than on wild oat (Figure 5B; *t* = 3.843, *p* = 0.0001). Fecundity was not significantly affected by larval host plant (Figure 5C; *t* = −0.52, *p* = 0.605) as females laid an average of 1202 and 1270 eggs/female when larvae were fed wheat and wild oat, respectively.

There was no significant effect of the larval host plant on adult longevity (Figure 5D; *F*_1,161_ = 1.127, *p* = 0.290). Males survived longer in the adult stage than females (Figure 5D; *F*_1,161_ = 30.739, *p* = 0.0001). The longevity of adult *M. separata* ranged from 15.07 to 15.20 days for females and 19.08 to 21.10 days for males.

## 4. Discussion

Weeds can play an important role in harboring pests in agroecosystems. Our findings suggest that the common weed, wild oat, associated with wheat fields is equally attractive for oviposition by *M. separata* and supports nearly as good larval development as wheat, aside from slightly slower development. This suggests that wild oat could harbor as many *M. separata* in wheat fields as the wheat crop and act as a refuge for *M. separata* when wheat fields are being treated with insecticides. Additionally, these findings can be applied to optimize field management strategies, ultimately improving wheat yield and quality by regularly eradicating weeds, identifying more wild hosts of pests, and exploring the impact of different weed species on pest habitats.

Inter and intraspecific variation in host plant quality affects various life history traits of herbivorous insects, including aspects of their development, reproduction, and survival. Therefore, herbivorous insects, especially mobile herbivores, actively choose their preferred host plants [13,14,15,27]. Studies on the relationships between oviposition choices and offspring performance in insects have demonstrated that most insects naturally prefer to oviposit on host plants that are optimal for the development of their offspring, commonly referred to as the ‘Mother knows best hypothesis’ [27,28]. Plant host selection is primarily guided by chemosensory cues emitted by plants, which are mainly detected by olfactory sensilla on insect antennae [29]. The chromosome-level genome of *M. separata* has been sequenced, and the complex genetic resources have been documented, paving the way for further studies on host plant selection or shifts at the molecular level [30]. In our study, the host plant choice of *M. separata* larvae matched better with larval development than the adult oviposition choice. Other studies have similarly shown that larvae can make better host plant choices for their own development than mothers [31,32]. This lack of alignment in host plant choice between mother and larvae may be because host plant choice by mothers can only be made based on volatile plant chemicals, plant surface allelochemicals and nutrients, and the physical properties of plants, whereas larvae can detect the nutritional value and allelochemicals within the plant tissue. Other factors such as plant cues that indicate protective qualities against biological and abiotic stressors may also play a role in their decision-making [33,34].

Location of a suitable feeding site is a critical part of the life of a newly hatched caterpillar [35]. Most newly hatched caterpillars, except for some leaf-mining species, have a prefeeding movement phase. This can involve local exploration of the leaf or nearby leaves, or longer-distance dispersal. For many newly hatched caterpillars, including the family Noctuidae, long-distance dispersal of newly hatched larvae is common. This dispersal is achieved by ballooning, a process in which the newly hatched larva lowers itself on a strand of silk and is carried by the wind [35]. Plant-related cues, such as odor, taste, and colors, determine the choices made by herbivorous insects, including newly hatched caterpillars [36,37,38,39]. In our study, *M. separata* larvae were given a choice between wheat and wild oat, which were placed 9 cm away from each other. For a newly hatched caterpillar, this is a significant distance. Although ballooning behavior was not involved in this host plant choice, our results clearly show that some olfactory or gustatory cue [40] was used by the larvae to overwhelmingly choose wheat over wild oat. This could have important implications because wild oat often grows within wheat fields, which means that newly hatched caterpillars that hatch on wild oat could disperse onto wheat. 

The longevity and reproductive capacity of Lepidoptera are primarily dependent on the amount of nutrients obtained during the larval stage [41]. However, numerous studies have demonstrated that adult diet can play an important role in somatic maintenance, especially reproductive output [28,42,43]. These studies have shown that adult lepidopterans can extend longevity and increase fecundity when they acquire threshold amounts of nutrients, mainly carbohydrates. We found that the pre-oviposition period was longer when *M. separata* larvae were fed wheat than wild oat, while adult longevity and fecundity did not significantly differ. Since we had provided a 10% honey solution to *M. separata* adults, it is possible that those reared on wild oat may have compensated for a deficient acquisition of larval nutrients by consuming more adult diet during the pre-oviposition period, which has been shown in some insects [34]. On the other hand, pupal weights were not significantly different between the two host plant treatments, and since pupal and adult weights are strongly correlated with fecundity [10], it may be unlikely that adult diet played a role in this experiment. 

## 5. Conclusions

We demonstrated that *M. separata* will indiscriminately lay eggs on wild oat and wheat when provided with both host plants, and their offspring can readily develop on wild oat with no significant difference in reproductive output compared to cultivated wheat. This is informative for wheat growers, as wild oat in and around their fields could easily harbor *M. separata* populations between wheat crop cycles and serve as a refuge when wheat crops are being treated with insecticides. Moreover, larvae may disperse from wild oat to cultivated wheat, as larvae have a strong preference for wheat over wild oat. Additionally, the mechanisms underlying these findings require further research, such as assessing plant quality and chemicals, testing various environmental factors, and evaluating gene functions contributing to the observed results. Our findings provide fundamental knowledge for developing better strategies for managing *M. separata* and wild oat.

## Figures and Tables

**Table 1:** Developmental times of *Mythimna separata* immature stages fed on leaves of either wheat or wild oat. Values are means ± SE. Asterisk indicates statistically significant difference between wheat and wild oat at *p* < 0.05.

Stage	Developmental Time (Days ± SE)	
	Wheat	Wild Oat
Egg	3.79 * ± 0.05	3.50 ± 0.06
First instar	3.12 ± 0.04	3.26 ± 0.07
Second instar	2.09 ± 0.03	2.06 ± 0.03
Third instar	2.05 * ± 0.03	2.17 ± 0.04
Fourth instar	2.11 ± 0.04	2.17 ± 0.05
Fifth instar	3.22 * ± 0.10	2.60 ± 0.06
Sixth instar	2.96 * ± 0.11	3.88 ± 0.11
Larval duration	15.53 * ± 0.10	16.13 ± 0.22
Pre-pupa	1.96 ± 0.12	2.31 ± 0.13
Pupa	11.70 * ± 0.11	14.21 ± 0.15
Sex ratio	1:0.98	1:1
Total immature stage	29.20 * ± 0.16	32.65 ± 0.24

## Data Availability

The data are available from the corresponding author upon reasonable request.

## References

[1] Sharma H.C., Sullivan D.J., Bhatnagar V.S. (2002). Population dynamics and natural mortality factors of the oriental armyworm, *Mythimna separata* (Lepidoptera: Noctuidae), in South Central India. Crop Prot..

[2] Jiang X., Luo L., Zhang L., Sappington T.W., Hu Y. (2011). Regulation of migration in *Mythimna separata* (Walker) in China: A review integrating environmental, physiological, hormonal, genetic, and molecular factors. Environ. Entomol..

[3] Koyama J., Matsumura M. (2019). Ecology and control of armyworm, *Mythimna separata* (Lepidoptera: Noctuidae) in Japan, with special reference to outbreak and migration. Jap. J. Appl. Entomol. Zool..

[4] Li B.L., Xu X.L., Ji J.Y., Wu J.X. (2018). Effects of constant and stage-specific-alternating temperature on the survival, development and reproduction of the oriental armyworm, *Mythimna separata* (Walker) (Lepidoptera: Noctuidae). J. Integr. Agric..

[5] Yang X.Q., Li X.R., Cang X.Z., Guo J.L., Shen X.J., Wu K.M. (2022). Influence of seasonal migration on the development of the insecticide resistance of oriental armyworm (*Mythimna separata*) to *λ*-cyhalothrin. Pest Manag. Sci..

[6] Cang X.Z., Zhao S.Y., Yang X.Q., Yuan H.B., Liu J., Liu D.Z., Yang X.M., Wu K.M. (2023). Migration monitoring and route analysis of the oriental armyworm *Mythimna separata* (Walker) in Northeast China. Agronomy.

[7] Zhao Y.Y., Su L., Li S., Li Y.P., Xu X.L., Cheng W.N., Wang Y., Wu J.X. (2018). Insecticide resistance of the field populations of oriental armyworm, *Mythimna separata* (Walker) in Shaanxi and Shanxi provinces of China. J. Integr. Agric..

[8] Xu C., Ji J.C., Zhu X.Z., Huangfu N.B., Xue H., Wang L., Zhang K.X., Li D.Y., Niu L., Chen R. (2023). Chromosome level genome assembly of oriental armyworm *Mythimna separata*. Sci. Data.

[9] Navasero M.M., Navasero J.M.M. (2022). Host plants of *Mythimna separata* (Walker) (Lepidoptera: Noctuidae) in the Philippines and inventory of world records. J. ISSAAS.

[10] Awmack C.S., Leather S.R. (2002). Host plant quality and fecundity in herbivorous insects. Annu. Rev. Entomol..

[11] McCormick A.C., Arrigo L., Eggenberger H., Mescher M.C., De Moraes C.M. (2019). Divergent behavioral responses of gypsy moth (*Lymantria dispar*) caterpillars from three different subspecies to potential host trees. Sci. Rep..

[12] Yang X.B., Liu T.X. (2009). Life history and life tables of *Bactericera cockerelli* (Homoptera: Psyllidae) on eggplant and bell pepper. Environ. Entomol..

[13] Niu Y.Q., Li X.W., Li P., Liu T.X. (2013). Effects of different cruciferous crops on the fitness of *Plutella xylostella* (Lepidoptera: Plutellidae). Crop Prot..

[14] Niu Y.Q., Sun Y.X., Liu T.X. (2014). Development and reproductive potential of diamondback moth (Lepidoptera: Plutellidae) on selected wild crucifer species. Environ. Entomol..

[15] Guo J.F., Zhang M.D., Gao Z.P., Wang D.J., He K.L., Wang Z.Y. (2021). Comparison of larval performance and oviposition preference of *Spodoptera frugiperda* among three host plants: Potential risks to potato and tobacco crops. Insect Sci..

[16] Erenstein O., Jaleta M., Mottaleb K.A., Sonder K., Donovan J., Braun H.J., Reynolds M.P., Braun H.J. (2022). Global trends in wheat production, consumption and trade. Wheat Improvement: Food Security in a Changing Climate.

[17] Shiferaw B., Smale M., Braun H., Duveiller E., Reynolds M.P., Muricho G. (2013). Crops that feed the world 10. Past successes and future challenges to the role played by wheat in global food security. Food Secur..

[18] Kaur R., Kaur S., Deol J.S., Sharma R., Kaur T., Brar A.S., Choudhary O.P. (2021). Soil properties and weed dynamics in wheat as affected by rice residue management in the rice-wheat cropping system in south Asia: A review. Plants.

[19] Holm L.G., Plucknett D.L., Pancho J.V., Herberger J.P. (1991). The World’s Worst Weeds. Distribution and Biology.

[20] Bajwa A.A., Akhter M.J., Iqbal N., Peerzada A.M., Hanif Z., Manalil S., Hashim S., Ali H.H., Kebaso L., Frimpong D. (2017). Biology and management of *Avena fatua* and *Avena ludoviciana*: Two noxious weed species of agro-ecosystems. Environ. Sci. Pollut. Res..

[21] Jäck O., Menegat A., Gerhards R. (2017). Winter wheat yield loss in response to *Avena fatua* competition and effect of reduced herbicide dose rates on seed production of this species. J. Plant Dis. Protect..

[22] Singh D., Rai L. (1977). Bionomics of rice cutworm, *Mythimna separata* (Walker). Entomon.

[23] Lv W.X., Xie X.C. (2022). Effect of fluctuating temperatures on development, reproduction and energy of oriental armyworm populations, *Mythimna separata*. J. Appl. Entomol..

[24] Wang G.P., Zhang Q.W., Ye Z.H., Luo L.Z. (2007). The role of nectar plants in severe outbreaks of armyworm *Mythimna separata* (Lepidoptera: Noctuidae) in China. Bull. Entomol. Res..

[25] Otuka A., Niiyama T., Jiang X.F. (2023). Possible source and migration pathway for early–summer immigrants of the Oriental armyworm, *Mythimna separata*, arriving in northern Japan. J. Integr. Agric..

[26] Jia J.W., Sun S.L., Kuang W.Q., Tang R., Zhang Z.F., Song C.M., Liu T.X., Jing X.F. (2019). A semi-synthetic diet and the potential important chemicals for *Mythimna separata* (Lepidoptera: Noctuidae). J. Insect Sci..

[27] Wang W.W., He P.Y., Zhang Y.Y., Liu T.X., Jing X.F., Zhang S.Z. (2020). The population growth of *Spodoptera frugiperda* on six cash crop species and implications for its occurrence and damage potential in China. Insects.

[28] Ning Z.H., Chen C., Cui B.S., Zhang Y.H., Xie T., Wang Q., Zhu Z.C., Bai J.H., Bouma T.J. (2020). ‘Mother knows best’: Maternal oviposition effects of a range-expanding insect herbivore degrade coastal wetlands by targeting juvenile foundation plant species. Land Degrad. Dev..

[29] Katte T., Shimoda S., Kobayashi T., Wada-Katsumata A., Nishida R., Ohshima I., Ono H. (2022). Oviposition stimulants underlying different preferences between host races in the leaf-mining moth *Acrocercops transecta* (Lepidoptera: Gracillariidae). Sci. Rep..

[30] Zhao H.B., Liu H.W., Liu Y.P., Wang C., Ma B.W., Zhang M.J., Zhang Y., Liu Y., Yang B., Wang S. (2023). Chromosome-level genomes of two armyworms, *Mythimna separata* and *Mythimna loreyi*, provide insights into the biosynthesis and reception of sex pheromones. Mol. Ecol. Resour..

[31] Winkler K., Wäckers F.L., Stingli A., van Lenteren J. (2005). *Plutella xylostella* (diamondback moth) and its parasitoid *Diadegma semiclausum* show different gustatory and longevity responses to a range of nectar and honeydew sugars. Entomol. Exp. Appl..

[32] Adar S., Roi D. (2018). Mother doesn’t always know best: Maternal wormlion choice of oviposition habitat does not match larval habitat choice. Behav. Process..

[33] Shikano I., Akhtar Y., Isman M.B. (2010). Relationship between adult and larval host plant selection and larval performance in the generalist moth, *Trichoplusia ni*. Arthropod-Plant Interact..

[34] Nandita N., Hansson B.S., Knaden M. (2024). Learning-based oviposition constancy in insects. Front. Ecol. Evol..

[35] Mevi-Schütz J., Erhardt A. (2005). Amino acids in nectar enhance butterfly fecundity: A long-awaited link. Am. Nat..

[36] Carrasco D., Larsson M., Anderson P. (2015). Insect host plant selection in complex environments. Curr. Opin. Insect Sci..

[37] Wang Y., Li R., Wang X., Liu X., Chen F. (2022). Elevated CO_2_ altered rice VOCs aggravate population occurrence of brown planthoppers by improving host selection ability. Biology.

[38] Hansson B., Stensmyr M. (2011). Evolution of insect olfaction. Neuron.

[39] Jing D., Prabu S., Zhang T., Bai S., He K., Wang Z. (2021). Genetic knockout and general odorant-binding/chemosensory protein interactions: Revealing the function and importance of GOBP2 in the yellow peach moth’s olfactory system. Int. J. Biol. Macromol..

[40] Bora D.S., Deka B., Sen A. (2013). Host plant selection by larvae of the Muga silk moth, *Anthe-Raea assamensis*, and the role of the antenna and maxillary palp. J. Insect Sci..

[41] Salgado A.L., Saastamoinen M. (2019). Developmental stage-dependent response and preference for host plant quality in an insect herbivore. Anim. Behav..

[42] Geister T.L., Lorenz M.W., Hoffmann K.H., Fischer K. (2008). Adult nutrition and butterfly fitness: Effects of diet quality on reproductive output, egg composition, and egg hatching success. Front. Zool..

[43] Pan Q.J., Ang Y., Shikano I. (2024). Effects of adult diet on the longevity, fecundity and ovarian development of the rice leaffolder, *Cnaphalocrocis medinalis*. Physiol. Entomol..

